# MINDFULNESS AND CAREGIVING EXPERIENCE IN ADRD CAREGIVERS

**DOI:** 10.1093/geroni/igac059.1772

**Published:** 2022-12-20

**Authors:** Magdalena Tolea, Iris Cohen, Simone Camacho, James Galvin

**Affiliations:** University of Miami Miller School of Medicine, Boca Raton, Florida, United States; University of Miami Miller School of Medicine, Boca Raton, Florida, United States; University of Miami Miller School of Medicine, Boynton Beach, Florida, United States; University of Miami School of Medicine, Miami, Florida, United States

## Abstract

Mindfulness (being present in the moment without judgement) has been linked to greater caregiver emotional health. Recent mindfulness-based interventions report improved coping skills, mood, and reduced stress in dementia caregivers. In this cross-sectional study of 141 ADRD caregivers, we assessed whether the relationship between caregiver mindfulness and caregiver experience varies by caregiver gender, relationship to patient (spouse-vs-child), etiology (AD-vs-LBD), or stage (MCI-vs-dementia). A stratified univariate analytic approach was used. Four mindfulness parameters (AMPS scale) were used: global score (GS), decentering (F1), positive (F2), and negative emotional regulation (F3). Outcomes included positive and negative appraisals of caregiving (PANAC), preparedness, care confidence, and depression. GS was linked to positive outcomes in male (rPANAC+=0.32/p=0.005), spouse caregivers (rPANAC+)=0.32/p=0.006 ) of ADRD patients regardless of etiology (rPANAC+=0.31/p=0.013 for AD; rconfidence=0.31/p=0.036 for LBD) and stage (rPANAC+=0.33/p=0.010 and rpreparedness=0.38,/p=0.008 for MCI; rPANAC+=0.29/p=0.011 and rconfidence=0.31/p=0.007 for dementia). Inverse relationships were observed with negative outcomes in male (rPANAC-=-0.46/p=0.002 and rdepression=-0.41/p=0.005), spouse caregivers (rPANAC-=-0.25/p=0.035 and rdepression=-0.30/p=0.009) of AD patients (rPANAC-=-0.25/p=0.043 and rdepression=-0.33/p=0.009) in early stages (rdepression=-0.41/p=0.001). F2 contributed to most relationships, with F3 and F1 significant in some but not all caregiver groups. Specifically, male spouse caregivers of AD patients regardless of stage may benefit from full-scope (F1-F3) programs while those of LBD patients from programs focused on improving emotional regulation (F2-F3). Wives of AD and LBD patients may in turn benefit from programs to improve positive emotional regulation (F2). Findings suggest that tailoring mindfulness-based interventions to specific caregiver groups may be effective in improving caregiver experience and mood.

